# Suppression of Allograft Rejection by Tim-1-Fc through Cross-Linking with a Novel Tim-1 Binding Partner on T Cells

**DOI:** 10.1371/journal.pone.0021697

**Published:** 2011-07-05

**Authors:** Liang Xiao, Zhi-ren Fu, Fang Liu, Lu-ding Zhang, Xiao-min Shi, Xiao-yun Shen, Zhi-jia Ni, Hong Fu, Rui-dong Li, Xue-tao Cao, Guo-shan Ding, Quan-xing Wang

**Affiliations:** 1 Department of Organ Transplantation, Shanghai Changzheng Hospital, Second Military Medical University, Shanghai, People's Republic of China; 2 National Key Laboratory of Medical Immunology, Second Military Medical University, Shanghai, People's Republic of China; University Paris Sud, France

## Abstract

Engagement of T-cell immunoglobulin mucin (Tim)-1 on T cells with its ligand, Tim-4, on antigen presenting cells delivers positive costimulatory signals to T cells. However, the molecular mechanisms for Tim-1-mediated regulation of T-cell activation and differentiation are relatively poorly understood. Here we investigated the role of Tim-1 in T-cell responses and allograft rejection using recombinant human Tim-1 extracellular domain and IgG1-Fc fusion proteins (Tim-1-Fc). *In vitro* assays confirmed that Tim-1-Fc selectively binds to CD4^+^ effector T cells, but not dendritic cells or natural regulatory T cells (nTregs). Tim-1-Fc was able to inhibit the responses of purified CD4^+^ T cells that do not express Tim-4 to stimulation by anti-CD3/CD28 mAbs, and this inhibition was associated with reduced AKT and ERK1/2 phosphorylation, but it had no influence on nTregs. Moreover, Tim-1-Fc inhibited the proliferation of CD4^+^ T cells stimulated by allogeneic dendritic cells. Treatment of recipient mice with Tim-1-Fc significantly prolonged cardiac allograft survival in a fully MHC-mismatched strain combination, which was associated with impaired Th1 response and preserved Th2 and nTregs function. Importantly, the frequency of Foxp3^+^ cells in splenic CD4^+^ T cells was increased, thus shifting the balance toward regulators, even though Tim-1-Fc did not induce Foxp3 expression in CD4^+^CD25^−^ T cells directly. These results indicate that Tim-1-Fc can inhibit T-cell responses through an unknown Tim-1 binding partner on T cells, and it is a promising immunosuppressive agent for preventing allograft rejection.

## Introduction

T-cell immunoglobulin mucin (Tim) proteins represent a newly discovered family of molecules that play critical roles in regulation of T helper cell 1 (Th1) and Th2 immune responses. Tim-1 protein belongs to a family of cell surface glycoproteins that modulate T-cell immune responses[1], [2], [3]. Ligation of Tim-1 molecule on T cells with Tim-4, a ligand for Tim-1 on mature dendritic cells (DCs) and macrophages, transmits a stimulatory signal in collaboration with the conventional TCR-dependent signal 1 and results in enhancement of T-cell proliferation, cytokine production and abrogation of tolerance[4], [5], [6], [7], [8], [9]. However, the mechanisms for immune regulation by Tim-1 and Tim-4 are more complex than initially expected. Recent studies showed that Tim-4-Ig may either stimulate or inhibit T-cell proliferation depending on its concentration[4]. Moreover, Tim-4-Ig was demonstrated to inhibit naive mouse CD4^+^ T-cell activation through a ligand other than Tim-1, and such an inhibitory effect of Tim-4-Ig was specific to naive T cells, and the effect disappeared in pre-activated T cells[10]. This suggests the possibility that the opposite effect of Tim-4-Ig on T-cell activation observed in the previous study[4] could be resulted from engagement with different receptors on T cells. On the other hand, anti-Tim-1 mAbs were also found to mediate either a stimulatory or an inhibitory effect on T-cell activation depending on their binding affinity to Tim-1[11], [12]. Thus, further elucidation of the role of Tim-1 in regulating T-cell responses is highly important for developing novel therapeutic strategies targeting Tim-1 for the treatment of autoimmune diseases and allograft rejection.

To further investigate the role of Tim-1 in T cell responses and allograft rejection, we expressed and purified recombinant human Tim-1 extracellular region and IgG1 Fc tail fusion proteins (Tim-1-Fc). We show that Tim-1-Fc selectively bind to CD4^+^ effector T cells (Teffs), but not DCs or natural regulatory T cells (nTregs). Interestingly, Tim-1-Fc significantly inhibited anti-CD3/CD28-stimulated proliferation and activation of purified CD4^+^ T cells that do not express Tim-4. Such an inhibitory effect of Tim-1-Fc was associated with decreased phosphorylation of AKT and ERK1/2, but the proliferation of nTregs was not inhibited. Moreover, Tim-1-Fc also inhibited allogeneic mixed lymphocyte reaction (allo-MLR) in vitro. Treatment with Tim-1-Fc successfully prolonged allograft survival in a MHC-mismatched murine cardiac transplantation model, this was associated with impaired Th1 response and preserved Th2 and nTregs function. Importantly, the proportion of Foxp3^+^ cells in splenic CD4^+^ T cells was increased, thus shifting the balance toward regulators, although Tim-1-Fc didn't induce Foxp3 expression in CD4^+^CD25^-^ T cells directly. These data indicate that Tim-1-Fc suppresses alloimmune responses not by blocking Tim-1 engagement with Tim-4 on DCs as previously indicated[2], [4], [13], but by interacting with a novel unknown ligand that is expressed on Teffs.

## Materials and Methods

### Animals

Male C57BL/6 (B6, H-2^b^), BALB/c (H-2^d^) and C3H (H-2^k^) mice (aged 8–10 weeks) were purchased from Shanghai Bikai Laboratory Animal Technology CO. LTD (Shanghai, China). All animals were housed under specific pathogen-free conditions in a 12 h day/night rhythm with free access to food and water ad libitum, and received humane care in accordance with international guidelines and the national law. The animal protocol of this study was approved by the Animal Care and Use committee of the Second Military Medical University (Permit Number. 08–0086). All surgery was performed under sodium pentobarbital anesthesia, and all efforts were made to minimize suffering.

### Antibodies and reagents

Magnetic separation system and mouse CD4^+^, CD4^+^CD25^+^ regulatory T cells (Tregs) isolation kits were products of Miltenyi Biotech. FITC-labeled anti-CD4 mAbs, specific rat IgG isotype control mAbs, PE-labeled anti-CD25, CD69 and Tim-4 mAbs were purchased from BD PharMingen (San Diego, CA). Functional anti-Tim-4 mAb was from Biolegend. Mouse Treg staining Kit (Foxp3 FJK-16s APC; CD4 FITC; CD25 PE) and ELISA kits for measuring mouse IL-2, IFN-γ, IL-4 and IL-10 were purchased from eBioscience. Human IgG1 (hIgG1) was from Calbiochem. Recombinant human TGF-β_1_ was product of PeproTech. The primary antibody (Rabbit) directed against p-AKT, p-ERK1/2 and ERK1/2 were purchased from Cell Signaling (Beverly, MA, USA).

### Preparation of recombinant Tim-1-Fc

Extracellular human Tim-1 cDNA was spliced with hinge-CH2-CH3 domains of IgG1 cDNA and cloned into the pcDNA3.1 mammalian expression vector (Invitrogen, Carlsbad, CA) containing a mouse Igκ secretion signal. Then the recombinant plasmid was transfected into CHOK1 cells to establish a stable line expressing Tim-1-Fc. Tim-1-Fc proteins were purified from CHOK1 cell culture supernatants with a Ni^2+^ affinity column (Qiagen, Valencia, CA, USA). Protein purity was assessed by Coomassie Blue staining after SDS-PAGE analysis using a 4∼20% Tris/glycine gradient gel. Immunoblot analysis was performed with mouse anti-human IgG1 Fc mAb (ARP American Research Products, Inc.) and rat anti-mouse mAb conjugated to horseradish peroxidase (Invitrogen), followed by enhanced chemiluminescence (Amersham Biosciences, Piscataway, NJ, USA). The molecular weight of Tim-1-Fc was about 62KD (Figure S1). Protein concentrations were determined using the Bradford reagent (Bio-Rad, Hercules, CA, USA). Endotoxin was then tested using the end-point colorimetric method (Cambrex Bio Science Walkersville, Walkersville, MD, USA) and found to have <1EU/µg.

### Preparation of bone marrow-derived DCs and splenic T cells

BALB/c (H-2^d^) mouse bone marrow-derived dendritic cells (DCs) were prepared as described[14]. CD4^+^ T cells were purified using the magnetic separation system according to the manufacturer's recommendations. Briefly, mouse spleen cells were prepared, incubated with CD4-microbeads for 20 min at 4°C, and then isolated by positive selection over a magnetic column. For isolation of CD4^+^CD25^−^ and CD4^+^CD25^+^ T cells[15], [16], mouse spleen cells were first depleted of CD4-negative cells by MACS separation system using a cocktail of biotin-conjugated antibodies against CD8 (Ly-2), CD11b (Mac-1), CD45R (B220), CD49b (DX5), and Ter-119 in combination with anti-biotin microbeads. The purified CD4^+^ spleen cells were then used to isolate CD4^+^CD25^−^ and CD4^+^CD25^+^ cells by positive selection using CD25-PE and anti-PE microbeads. The purity of all isolated T cell subsets was >95% as determined by flow cytometry (FCM).

### Measurement of Tim-1-Fc binding activity to T cells and DCs

Unstimulated or stimulated DCs, CD4^+^CD25^−^ and CD4^+^CD25^+^ T cells were incubated with 5 µg/ml Tim-1-Fc or hIgG1 and marked by mouse anti-human IgG1 Fc-FITC (Beckman Coulter), followed by FCM. Besides, anti-Tim-4 mAbs (10 µg/ml), which has been demonstrated to be able to block the binding of mouse Tim-1-Ig to Tim-4 (Figure S2) was added.

### T cells activation, proliferation and division assays

Ninety-six-well flat bottom Nunc plates (Rochester, NY, USA) were pre-coated with 2 µg/ml of anti-mouse CD3 mAb and 1 µg/ml of anti-mouse CD28 mAb. After washed with PBS, CD4^+^ T cells (2×10^5^/well) were plated in RPMI1640 medium supplemented with 10% FBS and phosphate buffered saline (PBS) or hIgG1 control or Tim-1-Fc (at a different final concentration of 1, 5 or 10 µg/ml), and incubated in 5% CO_2_ at 37°C for 72 h. ^3^H thymidine (1 µCi/well, Amersham Pharmacia Biotech, UK) was added to the culture for the final 18 h and T cell proliferation was measured by ^3^H thymidine incorporation using a liquid scintillation counter (Wallac, Turku, Finland). The supernatants were collected for cytokine detection. For cell division measurement, freshly isolated CD4^+^ T cells were labeled with CSFE (Molecular Probes, Eugene, OR, USA) according to the manufacturer's instructions, cultured in anti-CD3 and anti-CD28 mAbs-pre-coated plate as described above, and then analyzed by FCM 72 h later. To determine the effect of Tim-1-Fc on nTregs, purified CD4^+^CD25^+^ T cells (2×10^5^/well) were plated in the anti-CD3 and anti-CD28 mAbs (100 ng/ml for each) pre-coated plate in the presence of IL-2 (200 IU/ml)[17] with or without Tim-1-Fc at different final concentrations (1, 5 or 10 µg/ml). Then the proliferation and Foxp3 expression of nTregs were analyzed by ^3^H thymidine incorporation and SYBR green real-time polymerase chain reaction (RT-PCR).

### Measurement of Tim-4 expression on T cells

Tim-4 expression on freshly isolated and activated CD4^+^ T cells was assessed by FCM and RT-PCR. In FCM analysis, T cells were stained with anti-mouse Tim-4-PE or specific FITC-labeled rat IgG2a isotype control. For RT-PCR analysis, total RNA was isolated from purified CD4^+^ T cells and the expression of Tim-1, Tim-3 and Tim-4 was tested as described[18]. The primers used were presented in Table S1.

### Tim-4 antibody blocking assay

CD4^+^ T cells (2×10^5^/well) were cultured in RPMI1640 medium containing anti-CD3 and anti-CD28 mAbs and different concentration of anti-Tim-4 mAb (1, 5, 10 µg/ml) or PBS or control rat IgG1, with or without Tim-1-Fc (5 µg/ml). T-cell proliferation was measured 72 h later by ^3^H thymidine incorporation as described above. The supernatants were collected for cytokine detection.

### MLR assays

B/6 mouse CD4^+^ T cells (2×10^5^/well) were co-cultured with BALB/c mouse DCs at the indicated ratios with or without Tim-1-Fc (0, 5, 10 µg/ml) in 5% CO_2_ at 37°C for 72 h. T-cell proliferation was measured by ^3^H thymidine incorporation as described above. MLR supernatants were collected for cytokine detection.

In the experiments measuring secondary MLR responses, B/6 splenic CD4^+^ T cells (2×10^5^/well) were first incubated with irradiated (30Gy) BALB/c mouse DCs at a fixed stimulator:responder cell ratio (1∶10) in the presence or absence of Tim-1-Fc (0, 5, 10 µg/ml) (primary MLR). After three days, residual cells were incubated with FITC-conjugated anti-H-2^d^ mAb (BD Pharmingen), and H-2d^+^ BALB/c cells were removed by the MACS using anti-FITC-microbeads (Miltenyi Biotech). The recovered B/6 T cells were rested in culture medium for a further two days and then restimulated with irradiated (30Gy) splenocytes from BALB/c or C3H mice for 72 h (secondary MLR). T-cell proliferation was measured by ^3^H thymidine incorporation as described above.

### Signal pathways triggered by Tim-1-Fc exploring

CD4^+^ T cells were stimulated by incubation with anti-CD3 (2 µg/ml) and anti-CD28 (1 µg/ml) mAbs together with hIgG1 (10 µg/ml) or Tim-1-Fc (10 µg/ml) for indicated time. The cells were collected and lysed as described[19]. Briefly, twenty micrograms of each sample was subjected to SDS-PAGE and electrophoretic transfer to nitrocellulose membranes. Membranes were immunoblotted with p-AKT, p-ERK1/2, or total ERK1/2 antibodies. The bound antibodies were detected by appropriate HRP-conjugated anti-rabbit IgG. Quantification of protein levels were performed by densitometric analysis.

### Heart transplantation

Cervical heterotopic heart transplantation from BALB/c donors into B/6 mice was performed as described[20]. Recipient mice were injected intraperitoneally daily with 10 mg/kg of Tim-1-Fc or hIgG1, or equal volume of PBS at day 0–6 post-operation (POD 0–6). The contraction of heart grafts was monitored daily by two independent observers without prior knowledge of the treatment protocol. The complete cessation of cardiac contraction was defined as the endpoint.

### Histopathological examination and RT-PCR assays in allografts

Cardiac grafts were harvested on POD 7 and fixed in 10% formalin and embedded in paraffin. Sections were cut at 4 µm, and were counterstained for 1 min with hematoxylin eosin. Intragraft expression of IL-2, IFN-γ, IL-4, IL-10, CD11b, CD3 and Foxp3 mRNA were analyzed using RT-PCR as described[18]. The primers used were presented in Table S1. Transcript levels were calculated according to the 2^−ΔΔCt^ method[21].

### Statistical analysis

All values were expressed as means ± SD. For all data, normal versus non-normal distribution was assessed using the Shapiro-Wilk test and by visual examination of histograms. Homogeneity of variances was assessed by Levene test. For comparison of more than two variants with a normal distribution, one-way ANOVA analysis was used and followed by post hoc pair-wise comparisons using the Bonferroni correction. For comparison of more than two variants with a non-normal distribution, the Kruskal-Wallis test was used and followed by post hoc pair-wise comparisons using the Mann-Whitney test[22]. All of the above tests were performed using SPSS13.0 software. A *P* value of <0.05 was considered to be statistically significant.

## Results

### Tim-1-Fc binds to Teffs but not nTregs or DCs through a novel ligand other than Tim-4

**Figure 1:**
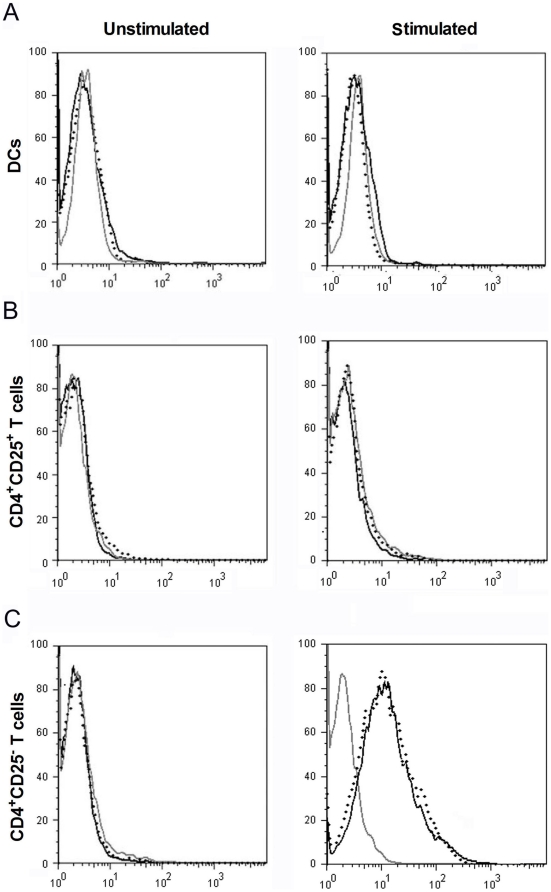
Expression of a binding partner for Tim-1 on CD4^**+**^ effector T cells. Unstimulated or 72 h LPS (100 ng/ml)-stimulated DCs (**A**), anti-CD3, anti-CD28 mAbs and IL-2 (200 IU/ml)-stimulated CD4^+^CD25^+^Tregs (**B**) and anti-CD3 (2 µg/ml), CD28 (1 µg/ml) mAbs-stimulated CD4^+^CD25^−^ T cells (**C**) were stained with 5 µg/ml control human IgG1 (gray line) or Tim-1-Fc (black line), followed by anti-human IgG1 Fc-FITC and subjected to FCM analysis. Anti-Tim-4 mAb (10 µg/ml, dotted line) were added to block the possible Tim-1-Tim-4 interaction.

To identify the binding activity of Tim-1-Fc to different mouse cell types, BM derived DCs, nTregs and CD4^+^CD25^−^T cells were prepared or isolated from B6 mice. There was no detectable binding of Tim-1-Fc to DCs or nTregs, even after activation (Figure 1A and B). However, upon stimulation with anti-CD3 and anti-CD28 mAbs for 72 h, CD4^+^CD25^−^ T cells showed a distinct peak shift compared with the hIgG_1_ control and this binding could not be abolished by anti-Tim-4 mAb (Figure 1C). These results suggested Tim-1-Fc selectively binds to CD4^+^ Teffs through a novel ligand other than Tim-4.

### Tim-1-Fc inhibits the activation and proliferation of Teffs but not nTergs

**Figure 2:**
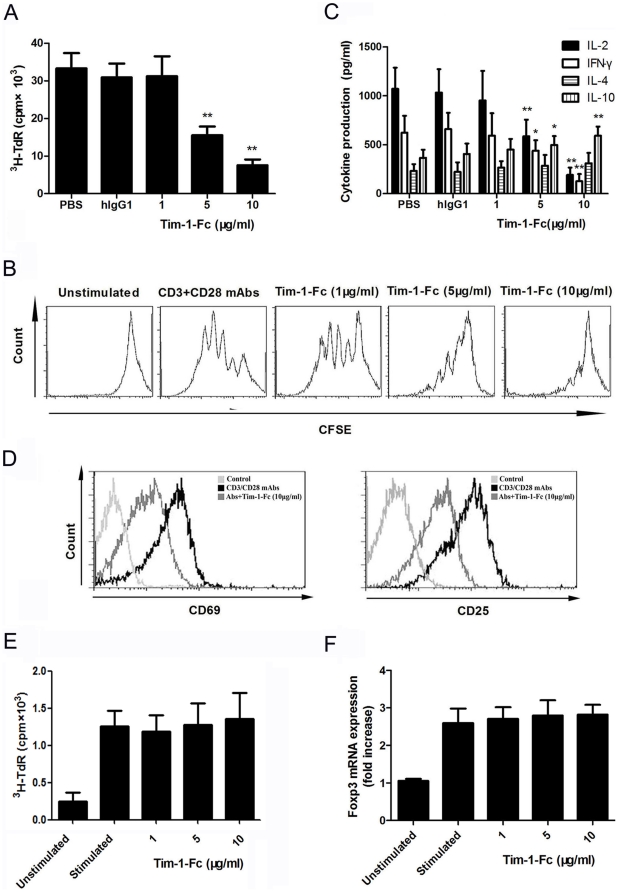
Tim-1-Fc inhibits the activation and proliferation of Teffs but not nTergs. Splenic CD4^+^ T cells (2×10^5^/well) were purified from C57BL/6 mice and were stimulated by anti-CD3 (2 µg/ml) and anti-CD28 (1 µg/ml) mAbs in the presence of Tim-1-Fc (1, 5, 10 µg/ml) or control PBS or hIgG1 for 72 h. Proliferation was measured by ^3^H thymidine uptake (**A**) and CSFE (**B**). Supernatants were obtained for cytokine ELISA (**C**). CD4^+^ T cells (2×10^5^/well) were stimulated with anti-CD3, anti-CD28 mAbs or together with Tim-1-Fc (10 µg/ml) in 96-well plates for 48 h, then were harvested to stain with anti-CD69, CD25-PE and analyzed by FCM. Data were representative of three experiments (**D**). Freshly isolated CD4^+^CD25^+^ regulatory T cells were stimulated with anti-CD3, anti-CD28 mAbs (100 ng/ml for each) and IL-2 (200 IU/ml) in the presence of Tim-1-Fc (1, 5, 10 µg/ml) for 72 h, the proliferation and Foxp3 expression of nTregs were assessed by ^3^H thymidine incorporation (**E**) or RT-PCR (**F**). *P<0.05, **P<0.01 in comparison with controls.

To further determine the effect of Tim-1-Fc on Teffs and nTregs, we compared the proliferation of purified CD4^+^ T cells in response to stimulation by anti-CD3 and anti-CD28 mAbs in the presence or absence of Tim-1-Fc. Interestingly, we found that Tim-1-Fc at a concentration of 5 µg/ml was able to significantly inhibit the proliferation of CD4^+^ T cells, and more pronounced at concentration of 10 µg/ml, compared with PBS or control hIgG1 treatment (Figure 2A). Dose-dependent inhibition of CD4^+^ T cell division by Tim-1-Fc was confirmed by FCM of CFSE dilutions (Figure 2B). Furthermore, Tim-1-Fc treatment significantly suppressed the production of IL-2 and IFN-γ, while increased the production of IL-10 (Figure 2C).

Expression of CD69 and CD25 are closely related to T cells activation. To analyze whether Tim-1-Fc inhibit T cell activation, the expression of CD69 and CD25 were measured using FCM. Stimulation of CD4^+^ T cells with anti-CD3 and anti-CD28 mAbs resulted in high expression of CD69 and CD25 on the majority of T cells. However, CD69 and CD25 expression was markedly inhibited in the presence of Tim-1-Fc (Figure 2D). Together, these results provide strong evidence that Tim-1-Fc can also inhibit Teffs responses.

Previous study has shown that immunostimulatory Tim-1-specific antibody (3B3) deprograms Tregs and prevents transplant tolerance[12], while immunosuppressive Tim-1-specific antibody (RMT1-10) enhances the function of nTregs and inhibits cardiac allograft rejection in mice[23]. We thought it is important to determine the influence of Tim-1-Fc on nTregs. Thus naïve CD4^+^CD25^+^ T cells were isolated and cultured with anti-CD3 and anti-CD28 mAbs plus IL-2 in the presence or absence of Tim-1-Fc for three days. Unlike the CD4^+^ T cells, addition of Tim-1-Fc into the cultures did not inhibit nTregs proliferation (Figure 2E) or Foxp3 expression (Figure 2F).

### Tim-1 cross-linking with a novel ligand on T cells other than Tim-4

**Figure 3:**
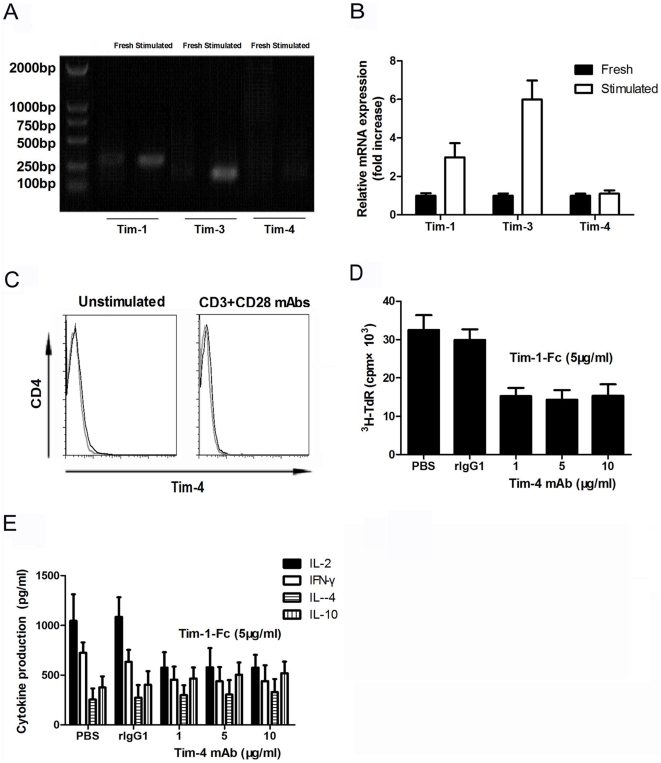
Tim-4 is not expressed by mouse T cells. Freshly isolated or anti-CD3 (2 µg/ml), anti-CD28 (1 µg/ml) mAbs activated CD4^+^ T cells were tested by RT-PCR (**A, B**) and stained with anti-mouse Tim-4-PE (black line) or specific isotype control (gray line) and subjected to FCM analysis. Data were representative of three experiments (**C**). CD4^+^ T cells were cultured with anti-CD3 and anti-CD28 mAbs in the presence of PBS or control rat IgG1 or Tim-1-Fc (5 µg/ml) together with anti-Tim-4 mAb (1, 5, 10 µg/ml) for 72 h. T cells proliferation and cytokines secretion were detected by ^3^H thymidine uptake (**D**) or ELISA (**E**).

Previous studies showed that Tim-4 is expressed on APCs, but not on either naive or activated/polarized T cells[4], [5], [6], [7], [8], [9]. To further rule out the possibility that the observed inhibitory effect of Tim-1-Fc on CD4^+^ T cells is mediated by engagement with Tim-4, we measured Tim-4 expression on CD4^+^ T cells by both RT-PCR and FCM. As shown in Figure 3A–C, neither freshly isolated nor activated CD4^+^ T cells showed detectable Tim-4. The data indicate that the observed inhibition of CD4^+^ T cell responses (Figure 2A–D) by Tim-1-Fc was unlikely mediated by Tim-4. In support of this, addition of anti-Tim-4 mAb failed to prevent Tim-1-Fc-mediated inhibition on proliferation or cytokine production of anti-CD3 and anti-CD28 mAbs-activated CD4^+^ T cells (Figure 3D and E). These results further indicate that Tim-1-Fc can inhibit CD4^+^ T cell responses by mechanisms independent of Tim-4. The data also implicate the presence of a novel Tim-1 ligand on CD4^+^ T cells.

### Tim-1-Fc suppresses allo-MLR and induces T cell hyporesponsiveness

**Figure 4:**
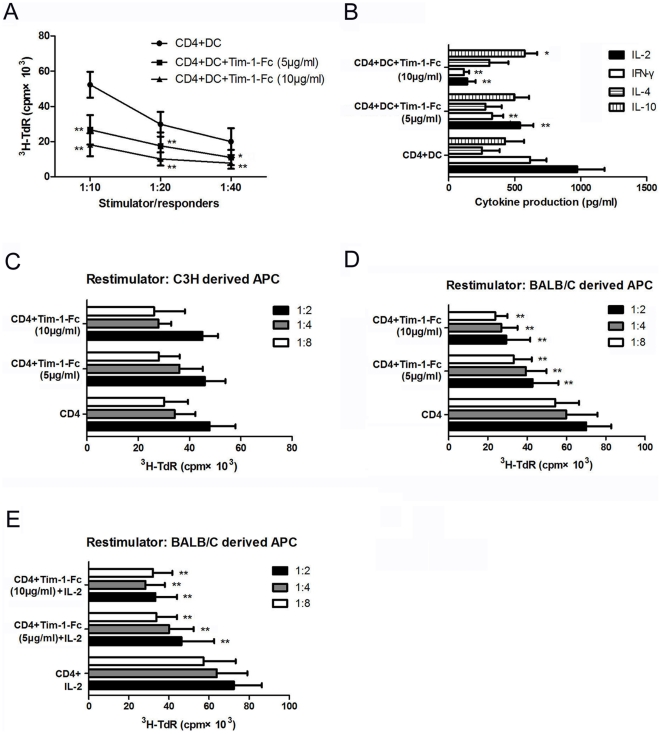
Tim-1-Fc inhibits allo-MLR and induces T cell hyporesponsiveness. CD4^+^ T cells (2×10^5^/well) were cocultured with BALB/C derived DCs at indicated ratios (1∶40, 1∶20, 1∶10) for 72 h, Tim-1-Fc (5, 10 µg/ml) was added to the culture at the beginning. Cell proliferation and cytokines production were evaluated by ^3^H-thymidine incorporation (**A**) and ELISA (**B**). C57BL/6 splenic CD4^+^ T cells (2×10^5^/well) were incubated at a fixed stimulator:responder cell ratio (1∶10) with irradiated allogeneic DCs in primary MLR. After three days, DCs were removed by autoMACS selection of H-2^d^ cells. The CD4^+^ T cells were rested in culture medium for a further two days, and then restimulated with irradiated C3H (H-2^k^, third-party) splenocytes (**C**), BALB/c derived splenocytes (**D**), or plus 20 IU/ml IL-2 (**E**) for 72 h (secondary MLR) at different stimulator:responder cell ratio. Cell proliferation was determined by ^3^H thymidine incorporation. *P<0.05, ** *P*<0.01 from the group without Tim-1-Fc treatment.

We next assessed the inhibitory effect of Tim-1-Fc on CD4^+^ T cell alloresponses by in vitro MLR assay. Mature DCs from BALB/c mice potently provoked the proliferation of B/6 CD4^+^ T cells (Figure 4A). However, the proliferation was significantly inhibited by Tim-1-Fc in a dose-dependent manner. Tim-1-Fc also significantly inhibited the production of IL-2, IFN-γ, while increased the production of IL-10 (Figure 4B). These results demonstrate that Tim-1-Fc can effectively inhibit allo-MLR.

The ability of Tim-1-Fc to induce T cell hyporesponsiveness to alloantigen restimulation was investigated. CD4^+^ T cells (2×10^5^/well) from B/6 mice primed with BALB/c DCs in the presence or absence of Tim-1-Fc in primary MLR for 3 days, and the responses of primed CD4^+^ T cells to BLAB/c or C3H (third-party) were measured in secondary MLR. Primed B6 CD4^+^ T cells strongly proliferated in response to stimulation with C3H splenocytes, regardless of whether Tim-1-Fc was added in the primary MLR (Figure 4C). However, the secondary response to BALB/c splenocytes of B/6 CD4^+^ T cells primed in the presence of Tim-1-Fc was significantly reduced compared to those primed without Tim-1-Fc (Figure 4D), and could not be restored by addition of IL-2 (Figure 4E). These results indicate that Tim-1-Fc treatment induced donor specific T cell hyporesponsiveness in vitro.

### Tim-1-Fc inhibits AKT and ERK1/2 phosphorylation in mouse T cells

**Figure 5:**
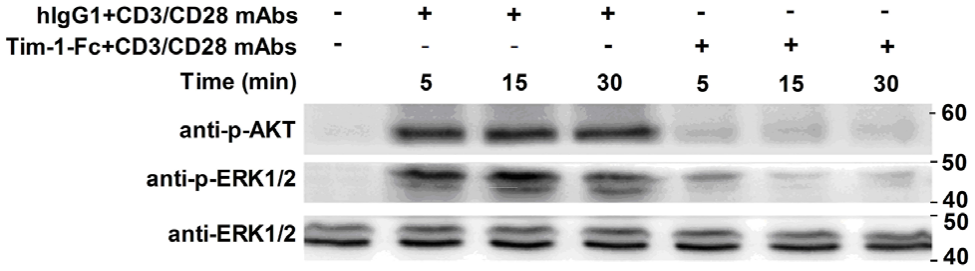
Tim-1-Fc inhibits AKT and ERK1/2 phosphorylation in mouse T cells. C57BL/6 mouse CD4^+^ T cells (2×10^6^/well) were stimulated by anti-CD3 (2 µg/ml) and anti-CD28 (1 µg/ml) mAbs plus 10 µg/ml Tim-1-Fc or control human IgG1 for indicated times. Whole-cell lysates were analyzed for kinase phosphorylation by western blotting. Data were representative of three separate experiments.

As extracellular signal-regulated protein kinase (ERK) kinases[24], [25] and AKT[26], [27] were important downstream signal molecular included upon TCR and CD28 engagement, we next assessed the effect of Tim-1-Fc treatment on cell signal transduction. As shown in Figure 5, the phosphorylation of AKT and ERK1/2 stimulated by anti-CD3 and anti-CD28 mAbs was significantly reduced by Tim-1-Fc treatment in CD4^+^ T cells. These results demonstrate that Tim-1-Fc affects both the MAP and AKT kinase signaling pathways, which may be at least one of mechanisms for the anti-proliferation activity of Tim-1-Fc.

### Tim-1-Fc prolongs allografts survival in a fully MHC-mismatched cardiac transplant model

**Figure 6:**
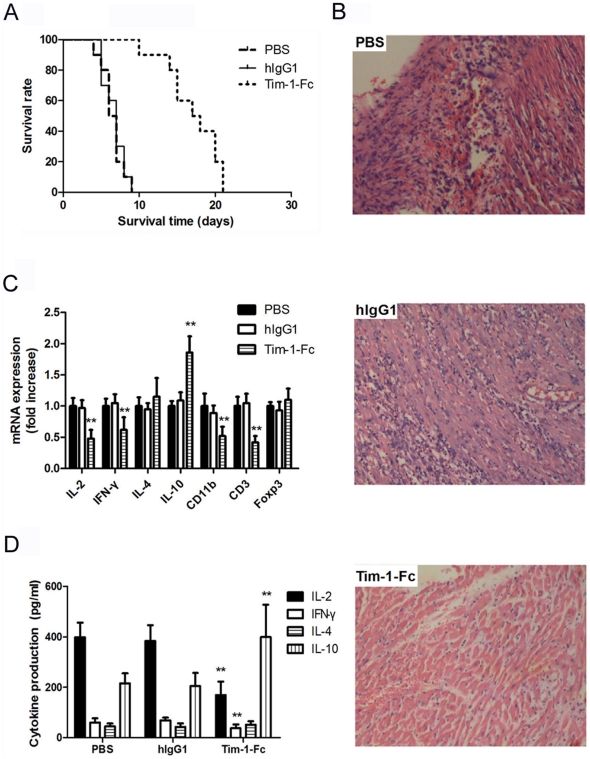
Tim-1-Fc prolongs cardiac allograft through inhibiting Th1 cytokines expression and leukocytes infiltration. Heterotopic heart grafts were transplanted from BALB/c mice into C57BL/6 recipients. The recipients were treated with PBS (n = 10), hIgG1 (10 mg/kg, n = 10) or Tim-1-Fc (10 mg/kg, n = 10) starting on post operation day (POD) 0 for seven consecutive days. Rejection was defined as cessation of a palpable impulse. Survival rates were compared using log-rank test (**A**). Hematoxylin eosin staining (**B**) of representative heart allografts harvested at POD 7 from mice treated with PBS, control hIgG1 or Tim-1-Fc (original magnification ×200). Cardiac allografts and blood were harvested on POD 7 from recipients. Intragraft expression of IL-2, IFN-γ, IL-4, IL-10, CD11b, CD3 and Foxp3 mRNA was measured by RT-PCR (**C**) and normalized against GAPDH. Serum levels of IL-2, IFN-γ, IL-4 and IL-10 were tested by ELISA (**D**). *P<0.05, ** *P*<0.01 in comparison with controls.

We then used a fully MHC-mismatched mouse heterotopic cardiac transplantation model to test the potential of Tim-1-Fc to prevent allograft rejection. BALB/c hearts were transplanted to B/6 recipients treated with daily injection of Tim-1-Fc (10 mg/kg/day) from POD 0–6. Control recipients were similarly treated with PBS or hIgG1. Recipients treated with Tim-1-Fc showed significantly prolonged allograft survival (17.1±1.1 days) compared to those treated with PBS (6.5±0.5 days; *P*<0.01) or hIgG1 (6.7±0.4 days; *P*<0.01, Figure 6A). Histological analysis of heart grafts at POD 7 revealed severe mononuclear cell infiltration and myocardial necrosis in the control groups, whereas almost normal tissue structure with minimal mononuclear cell infiltration was seen in Tim-1-Fc-treated mice (Figure 6B). These results indicate that Tim-1-Fc could effectively ameliorate histological damage and significantly prolong allograft survival.

### Tim-1-Fc inhibits leukocytes infiltration in the allograft and Th1 cytokines expression in the serum

RT-PCR analysis was performed to further understand the intragraft gene expression changes after Tim-1-Fc treatment, which significantly decreased intragraft CD11b (macrophages and neutrophils), CD3 (T cells) and IL-2, IFN-γ, while increased IL-10, and slightly increased IL-4 and Foxp3 gene expression compared with those in controls (Figure 6C). Moreover, Tim-1-Fc treatment significantly reduced the secretion of IL-2 and IFN-γ, while increased IL-10 levels in the serum (Figure 6D). Considering that Tim-1-Fc was able to inhibited Th1 cytokines production while promote Th2 responses in vivo, whether the administration of Tim-1-Fc results in increased production of antibody to allogenic heart should be assessed. However, we did not detect significant increase of alloantibody between hIgG1-Fc- and Tim-1-Fc-treated mice on POD 7 or 14 after transplantation (Figure S3).

### Tim-1-Fc induced hyporesponsiveness of CD4^+^ T cells to alloantigen

**Figure 7:**
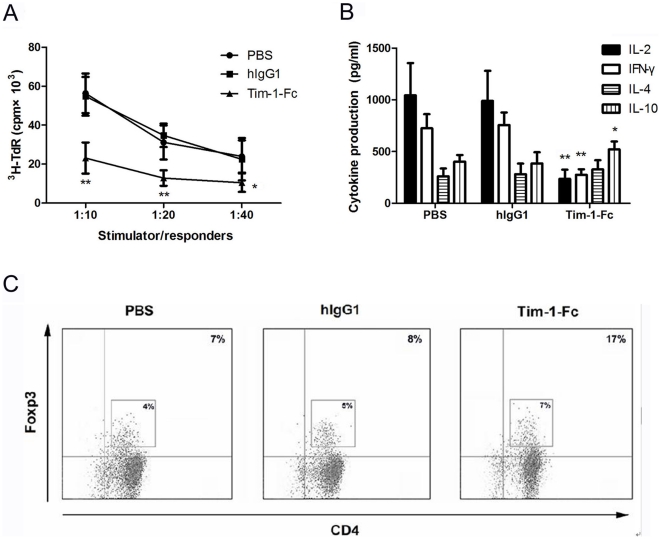
Tim-1-Fc induces hyporesponsiveness of CD4^**+**^ T cells to alloantigen and shifts the balance between Teffs and Tregs. CD4^+^ T cells (2×10^5^/well) purified from C57BL/6 recipient treated with PBS, hIgG1 and Tim-1-Fc for 7 days were cocultured with BALB/C derived DCs at indicated ratios (1∶40, 1∶20, 1∶10) for 72 h. Cell proliferation and cytokines production were evaluated by ^3^H-thymidine incorporation (**A**) and ELISA (**B**). *P<0.05, ** *P*<0.01 in comparison with controls. (**C**) The proportion of CD4^+^Foxp3^+^ T cells in C57BL/6 recipient splenic CD4^+^ T cells was determined by FCM analysis. The frequency of Foxp3^high^ and Foxp3^low^ cells was labled. Data were representative of three experiments.

Next, we isolated the splenic CD4^+^ T cells from either Tim-1-Fc treated or control B6 mice to explore their responses to alloantigen-specific DCs from BALB/C mice. As shown in Figure 7A, the T cell proliferation was significantly inhibited in Tim-1-Fc treated groups 72 h after restimulation, as compared to the controls. In concert with this, IL-2 and IFN-γ production was significantly inhibited, while the production of IL-10 was increased (Figure 7B). This data further documented that Tim-1-Fc induces hyporesponsiveness of CD4^+^ T to alloantigen (Figure 4).

### Tim-1-Fc shifts the balance between Teffs and Tregs

To further identify the influence of Tim-1-Fc on Foxp3 expression in recipient mice, CD4^+^ T cells were isolated from Tim-1-Fc treated mice or controls on POD 7 to test the percentage of CD4^+^Foxp3^+^ T cells. As shown in Figure 7C, Tim-1-Fc triggered a significant increase in the percentage of CD4^+^ T cells expressing a regulatory phenotype in the spleen (16.3%±2.3% vs. 8.2%±1.0%; *P*<0.01), but there was no significant difference in the Foxp3^high^ cells frequency between the control and treated mice. Given the inhibited proliferation capacity of CD4^+^ T compartment (Figure 2A), the increased frequency of Foxp3^low^ cells might be a consequence of decreased number of Teffs, but not the expansion of Tregs. However, these data strongly suggest Tim-1-Fc prolongs allograft survival by shifting the balance between Teffs and Tregs.

## Discussion

In the study presented herein, we demonstrated that Tim-1-Fc can selectively bind to Teffs through a novel ligand other than Tim-4. In vitro administrating Tim-1-Fc significantly inhibited the activation and proliferation of purified CD4 T cells, and their response to allogenic DCs. The inhibition of AKT and ERK1/2 phosphorylation is, at least, one of the mechanisms responsible for this APC/Tim-4-independent inhibitory effect. But Tim-1-Fc did not inhibit the proliferation or Foxp3 expression of nTregs. In vivo administration of Tim-1-Fc effectively prolonged cardiac allograft survival in a fully MHC-mismatched strain combination, this was associated with impaired Th1 response and preserved Th2 and nTregs function. Importantly, the proportion of Foxp3^+^ cells in splenic CD4^+^ T cells was increased, thus shifting the balance toward regulators. These results not only demonstrate the presence of an unidentified Tim-1 ligand capable of suppressing T cell activation upon biding to Tim-1-Fc, but also suggest the potential of Tim-1-Fc as a therapeutic agent for preventing allograft rejection.

Among T cells, Tim-1 was initially found to be predominantly expressed by Th2 cells, but accumulating data revealed that it is also expressed on murine and human Th1 cell lines, suggesting that Tim-1 may function as a global adjuvant of T-cell responses[28]. As a cognate ligand for Tim-1, Tim-4 was previously found to be expressed on mature, activated DCs and macrophages, but not on T cells, and Tim-4/Tim-1 engagement was shown to promote T-cell expansion and survival, suggesting that the Tim-1 pathway naturally functions as a potent T cell stimulator[5], [6], [7]. However, these may not always be the case, recent studies revealed that Tim-4 and Tim-1 could deliver either a positive or negative signal into T cells. Meyers et al[4] found that a higher dose (5 µg/ml) of Tim-4-Ig consistently led to an increase in T cell proliferation upon TCR ligation, whereas a lower concentration (1 µg/ml) of Tim-4-Ig inhibited T cell proliferation. This dose-dependent inhibition could be explained by several different mechanisms: It is possible that Tim-4 may engage another, high-affinity ligand on the surface of T cells that delivers a negative signal into T cells [10]. Alternatively, the effect of Tim-1 may depend on the ligand density and extent of engagement by Tim-4, in which Tim-1 delivers an inhibitory signal at lower levels of engagement and a positive signal at higher concentrations, similar to the agonist-antagonist phenomenon noted with altered peptide ligands[29]. Additionally, it was found that two monoclonal antibodies (clone 3B3 and clone RMT1-10) specific for the IgV domain of Tim-1 mediate opposite effects on T cell activation, in which the high avidity clone 3B3 enhances Th1 responses and prevented transplant tolerance induction, while the low avidity clone RMT1-10 prevents cardiac allograft rejection and induces Treg-dependent peripheral tolerance. Although these studies indicate that Tim-1 may serve as either a positive or a negative T cell costimulatory molecule depends on the ligand binding avidity, it remains unclear whether Tim-1 can also regulate T cell activation by engaging other ligands or receptors.

Using purified DCs, nTregs and CD4^+^CD25^−^ T cells, we observed that Tim1-1-Fc can selectively bind to Teffs and significantly inhibit CD4 T cells activation and proliferation in response to anti-CD3/CD28 mAbs. Although a previous study showed detectable Tim-4 mRNA in human CD4^+^ and CD8^+^ T cells[30], we could not detect Tim-4 on mouse CD4 T cells by either FCM or RT-PCR, which is consistent with many other studies[4], [6], [10], [31]. Moreover, anti-Tim-4 blocking antibodies could not prevent the effect of Tim-1-Fc, further supporting the possibility that the observed inhibition of CD4 T cells activation is Tim-4-independent and mediated by engaging with an unknown Tim-1 receptor that is expressed on CD4 T cells. The significant inhibition of AKT and ERK1/2 phosphorylation in CD4 T cells suggests that the AKT and MAP kinase signaling pathways are likely to be at least one of mechanisms for the anti-proliferative effect of Tim-1-Fc.

Previous study[4] has revealed that in vivo administration of Tim-1-Ig, a recombinant mouse Tim-1 extracellular domain and human IgG1-Fc fusion protein, resulted in higher levels of IL-4, IL-10 and lower levels of IL-2, IFN-γ in SJL/J mice. In our study, the impaired Th1 response may contribute to the prolonged cardiac allograft survival, however, we did not detect significant increase of alloantibody between hIgG1-Fc- and Tim-1-Fc-treated mice on POD 7 or 14 after transplantation. Previous studies have demonstrated that heart graft rejection in this model is T cell-dependent[32] and CD4^+^ T cells are both necessary and sufficient to mediate acute cardiac allograft rejection in mice[33], [34]. Although alloantibodies contribute to graft rejection, the production of alloantibodies is predominantly T cell-dependent. Thus, the graft rejection in Tim-1-Fc-treated mice is likely due to incomplete suppression of anti-donor T cell responses. Furthermore, Tim-1-Fc successfully shifted the balance towards regulators, as evidenced by the increased proportion of Foxp3 positive cells in recipients' spleens, which may also promote the immune tolerance[23]. In view that the inhibited proliferation capacity of effector CD4^+^ T compartment, the increased frequency of Foxp3^low^ cells might be a consequence of relatively decreased number of Teffs, but not the expansion of Tregs, as evidenced by the stable frequency of Foxp3^high^ cells. Besides, we also observed that stimulation of CD4^+^CD25^−^T cells with anti-CD3 and anti-CD28 mAbs in the presence of TGF-β1 significantly increased the frequency of CD4^+^Foxp3^+^ T cells, however, the addition of various concentrations of Tim-1-Fc had no additional effect, suggesting that Tim-1-Fc does not convert CD4^+^CD25^−^T cells into CD4^+^Foxp3^+^ Tregs directly (data not shown). Together, the data strongly suggest that Tim-1-Fc prolonged allograft survival by reducing the Teffs but not by increasing Tregs, however, the balance of Teffs/Tregs was shifted towards regulators.

In addition to Tim-4, phosphatidylserine and leukocyte mono-Ig–like receptor 5 (LMIR5)/CD300b were found to interact with Tim-1[35], [36]. Because phosphatidylserine is an apoptotic cell surface-specific epitope[37], it is unlikely to be involved in the inhibitory effect of Tim-1-Fc on T cells. In addition, LMIR5 is also unlikely to be responsible for Tim-1-Fc-mediated inhibition of CD4 T-cell activation in the absence of APCs, as this molecule is mainly expressed in myeloid cells, but not T cells[38], [39]. However, we detected other members of LMIR family such as LMIR1 and LMIR3, which are immunoreceptor tyrosine-based inhibitory motif-containing inhibitory receptors, are expressed by mouse T cells in the mRNA level (data not shown), whether they act as the endogenous inhibitory ligands for Tim-1 needs to be further explored.

In summary, we show that Tim-1-Fc mediates potent inhibition of T cells activation and significantly prolongs allograft survival in a MHC-mismatched murine cardiac transplantation model. This could be the selective binding of Tim-1-Fc to Teffs through a novel ligand other than Tim-4. These results further illustrate the importance of the Tim-1 and Tim-1-binding partner interaction in the regulation of immune responses and point to the possibility of developing immunosuppressive therapies to prevent allograft rejection.

## Supporting Information

Figure S1 **Western blot for Tim-1-Fc.** Immunoblot analysis for purified Tim-1-Fc was performed with mouse anti-human IgG1 Fc mAb and rat anti-mouse mAb conjugated to horseradish peroxidase, followed by enhanced chemiluminescence.
(TIF)

Figure S2 **Anti-Tim-4 inhibits ligand binding to Tim-4.** DCs from BALB/c mice were stimulated by LPS (100 ng/ml) and mouse Tim-1-Ig (eBioscience, 5 µg/ml) with or without anti-Tim-4 (10 µg/ml) for 3 days, then they were stained with anti-human IgG1 Fc-FITC and subjected to FCM analysis. Histograms represent Tim-1-Ig staining (black line) versus human IgG1 control (grey line) staining. Data are representative of two experiments.
(TIF)

Figure S3 **Production of donor-reactive Ab in C57BL/6 recipients of BALB/c cardiac allografts.** On POD 7 (A) and 14 (B) posttransplant, serum from PBS-, hIgG1- and Tim-1-Fc-treated (n = 6 for each) recipients was diluted, and each dilution was assayed by FCM for activity against naïve BALB/c splenocytes to determine the titer. Individual titers are shown with the mean and standard deviation represented by the bar. For each test day posttransplant, *P*>0.05 when comparing titers of donor-reactive Abs between Tim-1-Fc-treated and control mice.
(TIF)

Table S1 **Primers used in the study.**
(DOC)
