# Supplementary figures and images for: Suppression of Allograft Rejection by Tim-1-Fc through Cross-Linking with a Novel Tim-1 Binding Partner on T Cells
Source: PLoS One. 2011 Jul 5;6(7):e21697. doi: 10.1371/journal.pone.0021697 (PMC3130052; doi:10.1371/journal.pone.0021697)

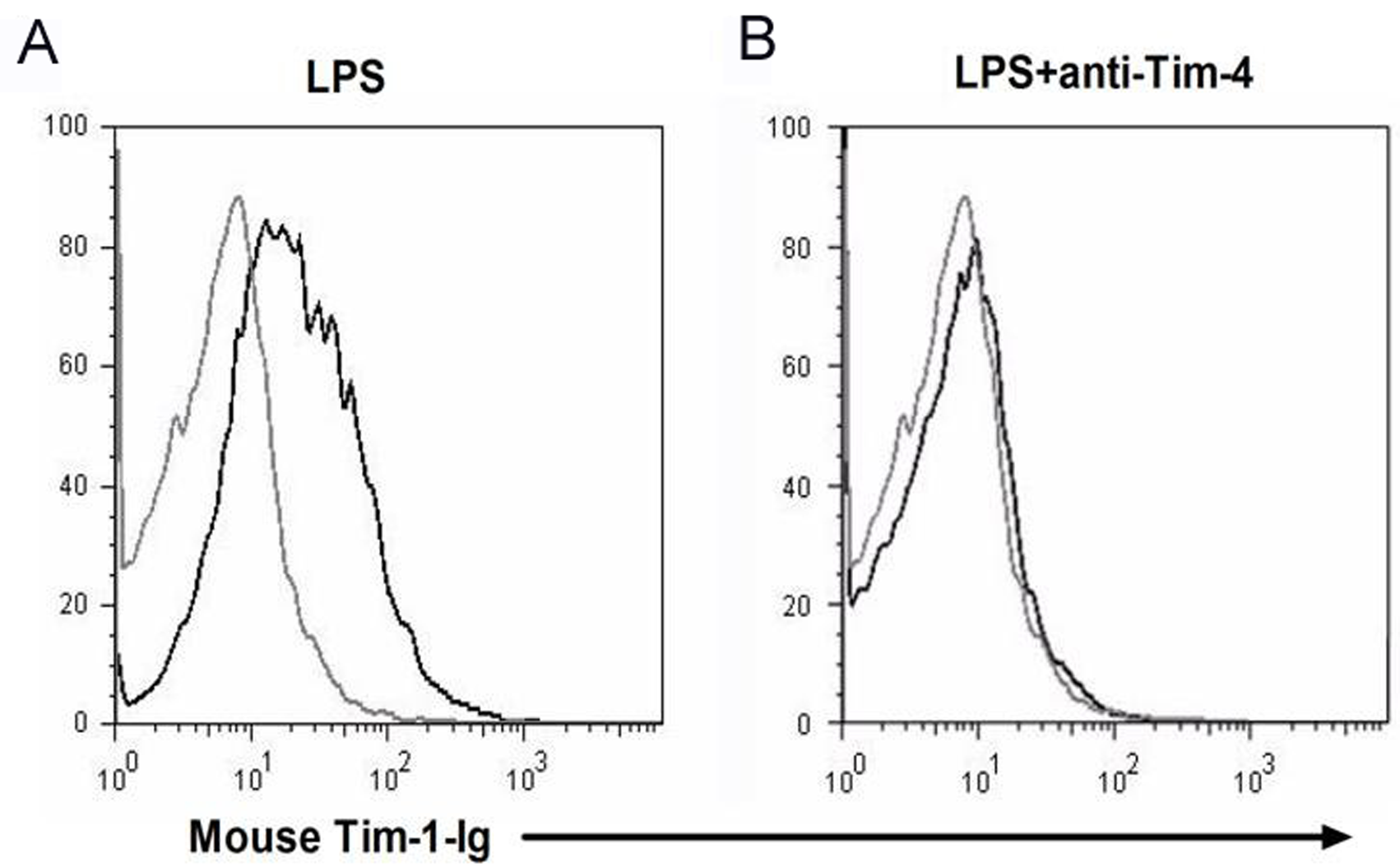

Supplement: Figure S2 — Anti-Tim-4 inhibits ligand binding to Tim-4. DCs from BALB/c mice were stimulated by LPS (100 ng/ml) and mouse Tim-1-Ig (eBioscience, 5 µg/ml) with or without anti-Tim-4 (10 µg/ml) for 3 days, then they were stained with anti-human IgG1 Fc-FITC and subjected to FCM analysis. Histograms represent Tim-1-Ig staining (black line) versus human IgG1 control (grey line) staining. Data are representative of two experiments. (TIF) [file pone.0021697.s002.tif]

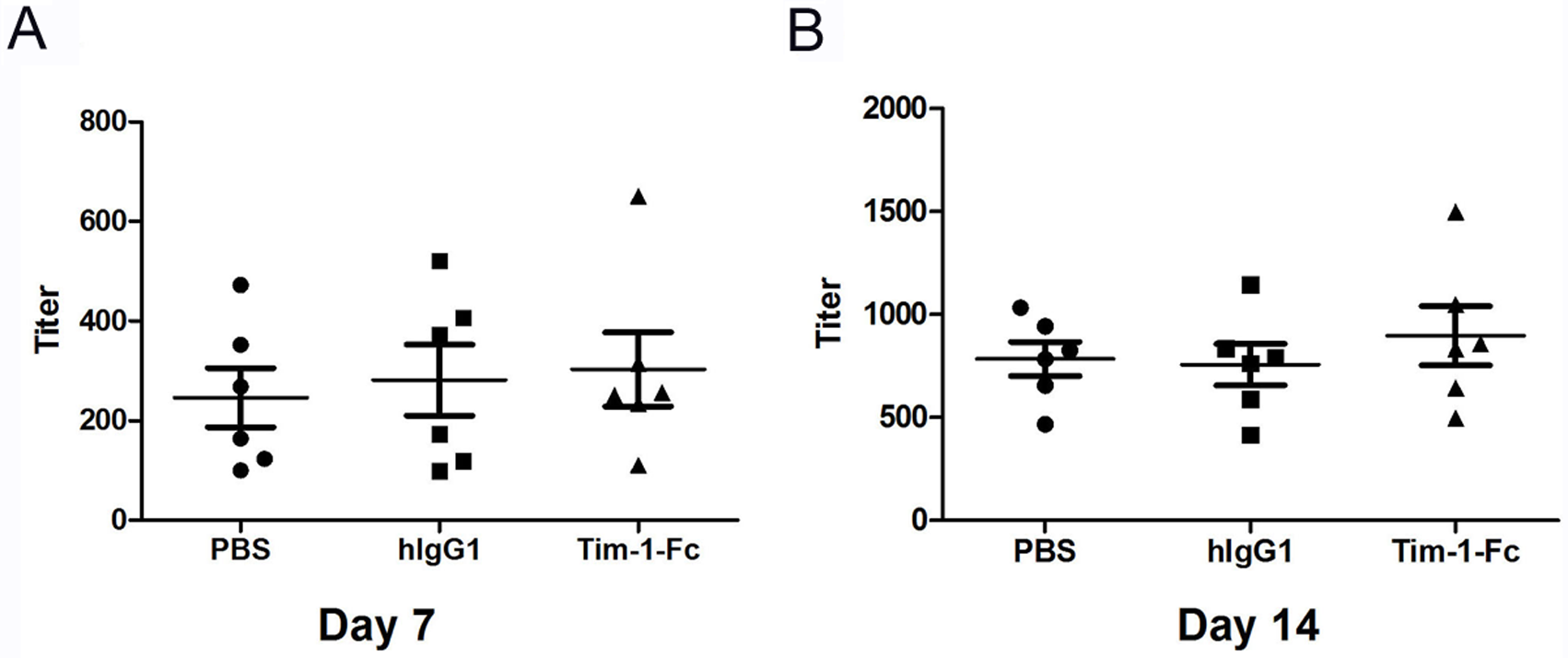

Supplement: Figure S3 — Production of donor-reactive Ab in C57BL/6 recipients of BALB/c cardiac allografts. On POD 7 (A) and 14 (B) posttransplant, serum from PBS-, hIgG1- and Tim-1-Fc-treated (n = 6 for each) recipients was diluted, and each dilution was assayed by FCM for activity against naïve BALB/c splenocytes to determine the titer. Individual titers are shown with the mean and standard deviation represented by the bar. For each test day posttransplant, P>0.05 when comparing titers of donor-reactive Abs between Tim-1-Fc-treated and control mice. (TIF) [file pone.0021697.s003.tif]
