# Supplementary material for: Suppression of Allograft Rejection by Tim-1-Fc through Cross-Linking with a Novel Tim-1 Binding Partner on T Cells
Source: PLoS One. 2011 Jul 5;6(7):e21697. doi: 10.1371/journal.pone.0021697 (PMC3130052; doi:10.1371/journal.pone.0021697)
Supplement: Table S1 — Primers used in the study. (DOC) [file pone.0021697.s004.doc]

**Supplemental Table 1. Primers used in the study.**

| IL-2 | sense: 5’-CCC AGG ATG CTC ACC TTC AA-3’, |
| --- | --- |
|  | antisense: 5’-CAT GCC GCA GAG GTC CAA-3’ |
| IFN-γ | sense: 5’-GAA CTG GCA AAA GGA TGG TGA-3’ |
|  | antisense: 5’-TGT GGG TTG TTG ACC TCA AAC-3’ |
| IL-4 | sense: 5’-GGT CTC AAC CCC CAG CTA GT-3’ |
|  | antisense: 5’-GCC GAT GAT CTC TCT CAA GTG AT-3’ |
| IL-10 | sense: 5’-GCT CTT ACT GAC TGG CAT GAG-3’ |
|  | antisense: 5’-CGC AGC TCT AGG AGC ATG TG-3’ |
| CD11b | sense: 5’-ATG GAC GCT GAT GGC AAT ACC-3’ |
|  | antisense: 5’-TCC CCA TTC ACG TCT CCC A-3’ |
| CD3 | sense: 5’-AGA GGG CAA AAC AAG GAG CG-3’ |
|  | antisense: 5’-AGA CTG CTC TCT GAT TCA GGC-3’ |
| Foxp3 | sense: 5’-CCC ATC CCC AGG AGT CTT G-3’ |
|  | antisense: 5’-ACC ATG ACT AGG GGC ACT GTA-3’ |
| Tim-1 | sense: 5’-GTT AAA CCA GAG ATT CCC ACA CG-3’ |
|  | antisense: 5’-TCT CAT GGG GAC AAA ATG TAG TG-3’ |
| Tim-3 | sense: 5’-TCA GGT CTT ACC CTC AAC TGT G-3’ |
|  | antisense: 5’-GGG CAG ATA GGC ATT TTT ACC A-3’ |
| Tim-4 | sense: 5’-TTG ATG ATT CAA GGC CAT CGT T-3’ |
|  | antisense: 5’-AGC TGG TGT CAG ATA AAG CCA-3’ |
| GAPDH | sense: 5’-ACC ACA GTC CAT GCC ATC AC-3’ |
|  | antisense: 5’-TCC ACC ACC CTG TTG CTG TA -3’ |
